# Pioglitazone Ameliorates Hippocampal Neurodegeneration, Disturbances in Glucose Metabolism and AKT/mTOR Signaling Pathways in Pentyelenetetrazole-Kindled Mice

**DOI:** 10.3390/ph15091113

**Published:** 2022-09-06

**Authors:** Nada El-Megiri, Yasser M. Mostafa, Amal Ahmed, Eman T. Mehanna, Mona F. El-Azab, Fatma Alshehri, Hadil Alahdal, Norhan M. El-Sayed

**Affiliations:** 1 Department of Pharmacology and Toxicology, Faculty of Pharmacy, Suez Canal University, Ismailia 41522, Egypt; 2 Department of Pharmacology & Toxicology, Faculty of Pharmacy, Badr University, Badr 11829, Egypt; 3 Department of Cytology and Histology, Faculty of Veterinary Medicine, Suez Canal University, Ismailia 41522, Egypt; 4 Department of Biochemistry, Faculty of Pharmacy, Suez Canal University, Ismailia 41522, Egypt; 5 Department of Biology, College of Sciences, Princess Nourah Bint Abdulrahman University, P.O. Box 84428, Riyadh 11671, Saudi Arabia

**Keywords:** epilepsy, pioglitazone, pentylenetetrazole, m-TOR, glucose metabolism, nerve growth factor

## Abstract

Disturbance of glucose metabolism, nerve growth factor (NGF) and m-TOR signaling have been associated with the pathophysiology of epilepsy. Pioglitazone (PGZ) is an anti-diabetic drug that shows a protective effect in neurodegenerative diseases including epilepsy; however, its exact mechanism is not fully elucidated. The present study aimed to investigate the potential neuroprotective effect of PGZ in pentylenetetrazole (PTZ) kindled seizure in mice. Swiss male albino mice were randomly distributed into four groups, each having six mice. Group 1 was considered the control. Epilepsy was induced by PTZ (35 mg/kg i.p.) thrice a week for a total of 15 injections in all other groups. Group 2 was considered the untreated PTZ group while Group 3 and Group 4 were treated by PGZ prior to PTZ injection at two dose levels (5 and 10 mg/kg p.o., respectively). Seizure activity was evaluated after each PTZ injection according to the Fischer and Kittner scoring system. At the end of the experiment, animals were sacrificed under deep anesthesia and the hippocampus was isolated for analysis of glucose transporters by RT-PCR, nerve growth factor (NGF) by ELISA and mTOR by western blotting, in addition to histopathological investigation. The PTZ-treated group showed a significant rise in seizure score, NGF and m-TOR hyperactivation, along with histological abnormalities compared to the control group. Treatment with PGZ demonstrated a significant decrease in NGF, seizure score, m-TOR, GLUT-1 and GLUT-3 in comparison to the PTZ group. In addition, improvement of histological features was observed in both PGZ treated groups. These findings suggest that PGZ provides its neuroprotective effect through modulating m-TOR signaling, glucose metabolism and NGF levels.

## 1. Introduction

Epilepsy is one of the most common neurological disorders affecting more than 60 million people globally. It is characterized by excessive neuronal firing, resulting in uncontrolled convulsions [1]. Although epilepsy can be well controlled in most patients through administration of antiepileptic drugs (AEDs), nearly 30% of patients develop resistance to the available medications [2]. The mechanisms of action of the existing antiseizure drugs focus mainly on regulating neurotransmitters and ion channels. Targeting novel mechanisms of action that are largely different from the current is important in order to develop treatment that is effective in refractory epilepsy cases [3]. The molecular basis of epileptogenesis is complex, and it is found to be a condition that is specific in several cases. Nevertheless some mechanisms appear to be common in many types of epilepsy. Abnormal activity of the mammalian target of rapamycin (mTOR) pathway was found to play an important role in epileptogenesis [4]. AMP-activated protein kinase (AMPK) activation and protein kinase B (AKT) hyperactivation could inhibit mTORC. A readout for mTOR activity is 4E-BP1 [4]. Many experimental models of acquired and genetic epilepsy where mTOR was found to be hyperactivated respond to mTOR inhibitors [5,6]. This reinforces the hypothesis that dysregulation of the mTOR pathway is pivotal for the development of epileptogenesis and epilepsy. In addition, a link between glycolysis and carbohydrate metabolism has been reported to contribute to the pathogenesis of epilepsy [7]. Nerve growth factor (NGF) has also been proven to be implicated in the development of epilepsy [8]. It was found that NGF can accelerate epileptogenesis. During seizure activity, increases in the level of NGF lead to further seizure development through mossy fiber sprouting [9].

Pioglitazone, a peroxisome proliferator-activated receptor (PPAR)-γ agonist [10], is a FDA-approved drug indicated for use in type 2 diabetes mellitus. The administration of PPAR-γ agonists have been found to provide neuroprotection and improve neurological functions in animal models of epilepsy [11] and, particularly, in numerous PTZ animal models [12,13]. The anti-epileptic effect of PGZ in those studies was revealed to be via its anti-inflammatory and anti-apoptotic effects. Hence, the current study aimed to investigate the neuroprotective effect of PGZ in a PTZ induced seizure model, focusing on its effect on glucose metabolism, the m-TOR pathway and NGF.

## 2. Results

### 2.1. Pioglitazone Reduced Mean Seizure Score

**Figure 1:**
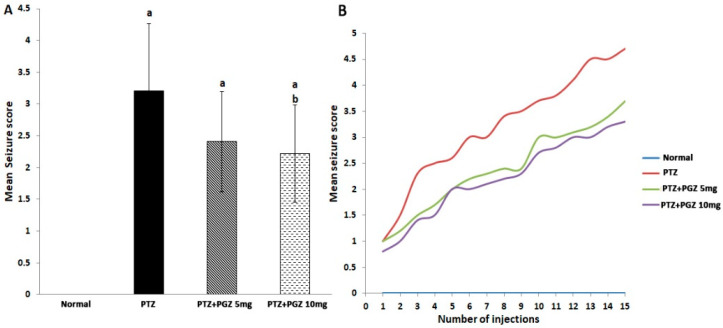
Pioglitazone reduced the mean seizure scores in PTZ-kindled mice. Induction of kindling was accomplished through injecting PTZ (35 mg/Kg, i.p.) thrice a week for five weeks and the intensity of seizure scores were assessed in accordance to Fischer and Kittner scoring scale. (**A**) Mean of seizure scores exhibited by mice over the fifteen injections of PTZ. Analysis of quantitative variables was done using one-way ANOVA followed by Bonferroni’s post-hoc test with *p* < 0.05 considered significant (n = 6). ^a^ significant difference from normal, ^b^ significant difference from PTZ group. (**B**) Time course of seizure scores demonstrated by mice after each PTZ injection. Analysis of quantitative variables was done using two-way ANOVA followed by Tukey LSD post-hoc test with *p* < 0.05 considered significant (n = 6). Data were expressed as means ± SD. PTZ: pentylenetetrazole, PGZ: pioglitazone.

In this study, kindling was achieved following fifteen injections of PTZ, as shown in Figure 1B. The mean of seizure scores in the PTZ group over fifteen injections was 3.2 ± 1 (Figure 1B). Analysis of mean seizure scores over 15 injections using 2-way ANOVA revealed a gradual increase in seizure score commencing from the third injection with PTZ (Figure 1B). Pre-treatment with PGZ reduced the mean seizure score in comparison to the PTZ group (Figure 1A). Importantly, only group 4, which was treated with a high dose of PGZ, demonstrated a significant decrease in seizure score in comparison to the PTZ group [F (3, 56) = 43.07, *p* < 0.05].

### 2.2. Pioglitazone Improved the Histological Features in the Hippocampus and Cortex of PTZ Epileptic Mice

**Figure 2:**
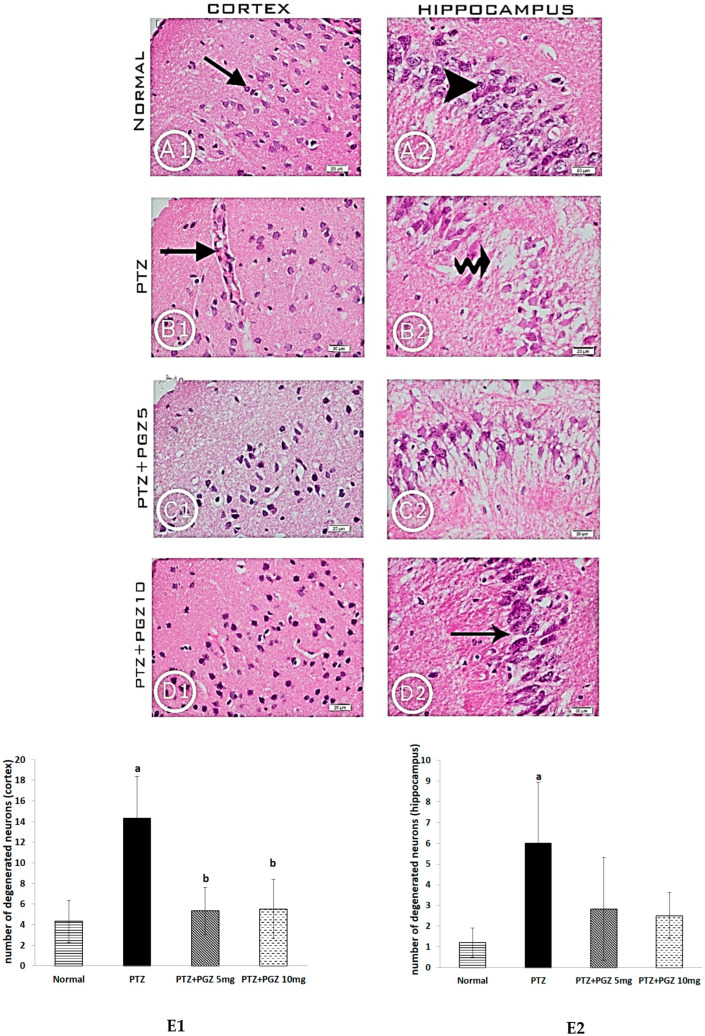
Pioglitazone improved the histopathological features of the cortex and hippocampus in PTZ-kindled mice. Photomicrographs of the sections of cerebral cortices (**A1**,**B1**,**C1**,**D1**) and area CA3 in the hippocampus (**A2**,**B2**,**C2,D2**) of the experimental groups stained with H&E. (**A1**,**A2**) Control group (**B1**,**B2**) PTZ-treated group (**C1**,**C2**) PTZ + PGZ (5 mg/kg p.o) and (**D1**,**D2**) PTZ + PGZ (10 mg/kg p.o). Cerebral cortices of control mice (**A1**) demonstrating basophilic neurons (arrow). PTZ-kindled mice (**B1**) showed shrunken neurons, light basophilic astrocytes and vacuolated neuropils with engorged blood capillaries (arrow). Mice treated with PGZ (5 mg/kg p.o) (**C1**) & (10 mg/kg p.o) (**D1**) showed neurons with normal appearance and dark basophilic astrocytes. Note that the blood capillaries were devoid of trapped red blood cells. Compared to the normal morphology of pyramidal neurons (arrowhead) in area CA3 of the hippocampus in the control group (**A2**), PTZ-kindled mice (**B2**) displayed shrinkage and degeneration of pyramidal cells (curved arrow). There was markedly less degeneration in the hippocampi of animals treated with 5 mg/kg PGZ and a further reduction of degenerated neurons with well-organized pyramidal cells (arrow) in animals treated with 10 mg/kg. (Scale bar 20 µm). Mean values of degenerative cell count in cortex (**E1**) and hippocampus (**E2**) data are expressed as mean ± SD. Analysis of quantitative variables was performed using one-way ANOVA followed by Bonferroni’s post-hoc test with *p* < 0.05 considered significant (n = 6). ^a^ significant difference from normal, ^b^ significant difference from PTZ group, ^c^ significant difference from low dose of PGZ. PTZ: pentylenetetrazole, PGZ: pioglitazone.

Figure 2 shows the photomicrographs for cortical and hippocampal sectors stained with H&E from different experimental groups. Cerebral cortices of the control group (Figure 2A1) displayed normal neurons, namely, vesicular, euchromatic nuclei and basophilic neurons surrounded by prominent nuclei of astrocytes, while the PTZ group (Figure 2B1) demonstrated shrunken neurons, light basophilic astrocytes and vacuolated neuropils. Capillaries with blood were observed in the PTZ group and noted with arrows. PGZ-treated groups (5 mg/kg p.o) (Figure 2C1) and (10 mg/kg p.o) (Figure 2D1) revealed unshrunk neurons and dark basophilic astrocytes. Sections of experimental groups treated by PGZ showed that the blood capillaries were devoid of trapped red blood cells. Sections of field CA3 in the hippocampus of the control group exhibited normal architecture (Figure 2A2) with vesicular nuclei of pyramidal cells. Sections from PTZ-treated mice (Figure 2B2) displayed a shrinkage of pyramidal cells, marked neural degeneration, with areas of paucity and indistinct nuclei. The group treated with PGZ (5 mg/kg p.o) (Figure 2C2) showed less distorted neurons with shrinkage of some cells. The group treated with PGZ (10 mg/kg p.o) (Figure 2D2) revealed a reduction of the degenerated neurons with vesicular nuclei, well organized pyramidal cells and loss of areas of paucity among the cells.

### 2.3. Pioglitazone Reduced NGF Levels

**Figure 3:**
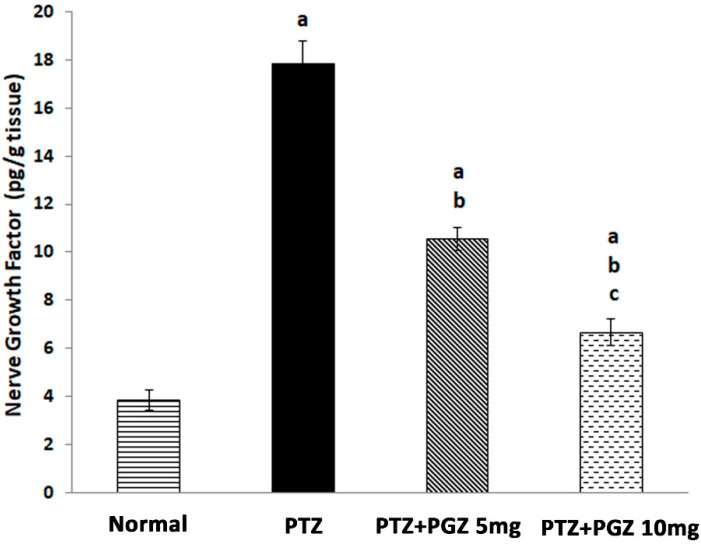
Pioglitazone reduced the hippocampal levels of nerve growth factor in PTZ-kindled mice. Mean values of nerve growth factor in the hippocampus, pg/g: picogram per gram. Data expressed as means ± SD. Analysis of quantitative variables was performed using one-way ANOVA followed by Bonferroni’s post-hoc test with *p* < 0.05 considered significant (n = 6). ^a^ significant difference from normal, ^b^ significant difference from PTZ group, ^c^ significant difference from low dose of pioglitazone. PTZ: pentylenetetrazole, PGZ: pioglitazone.

We observed a significant increase in NGF levels in the PTZ group in comparison to control (Figure 3). However, pre-treatment with PGZ significantly decreased NGF levels in comparison to the PTZ group [F (3, 20) =284.38, *p* < 0.05].

### 2.4. Pioglitazone Upregulated AKT/AMPK and Downregulated mTOR Signaling Pathways

**Figure 4:**
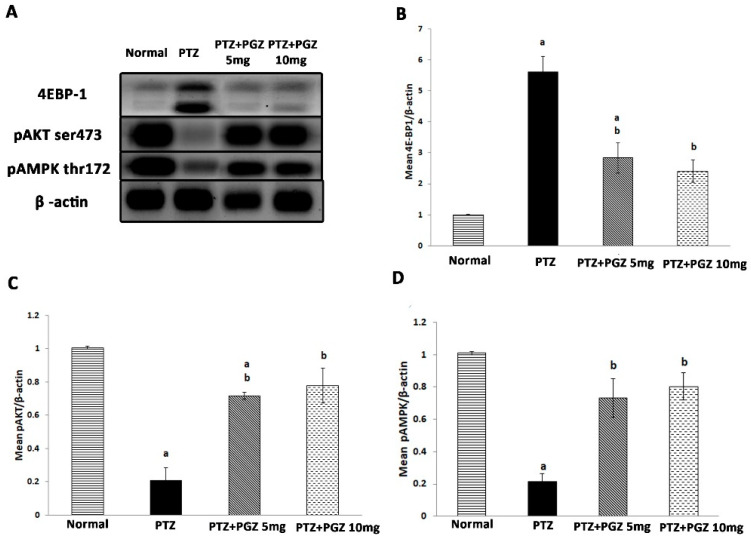
Pioglitazone upregulated hippocampal levels of pAKT and pAMPK while it decreased levels of 4EBP1 in PTZ-kindled mice. (**A**) Representative western blot result for 4EBP-1, pAKT and pAMPK expression in hippocampi from the different groups. β-actin served as a control for loading. Quantitative data of 4EBP1 (**B**), pAKT (**C**) and pAMPK (**D**) with respect to β-actin. Data were expressed as means ± standard deviation. Analysis of quantitative variables was performed using one-way ANOVA followed by Bonferroni’s post-hoc test with *p* < 0.05 considered significant (n = 4). ^a^ significant difference from normal, ^b^ significant difference from PTZ group. pAMPK: phosphorylated adenosine monophosphate-activated protein kinase, 4EBP1: eukaryotic translation initiation factor 4E-binding protein 1, PTZ: pentylenetetrazole, PGZ: pioglitazone.

We next evaluated the effect of PTZ for 15 days on the levels of pAMPK α (Thr172), pAKT (Ser473), and 4E-BP1 in the hippocampus by western blot (Figure 4). The intensity of pAMPK, pAKT, and 4EBP1 proteins were normalized against β-actin and quantified using Image J (NIH). Kindling with PTZ significantly decreased the expression of pAMPK [F (3, 8) = 23.93, *p* < 0.05] and increased 4E-BP1 [F (3, 8) = 29.94, *p* < 0.05], while, surprisingly, p AKT expression decreased [F (3, 8) = 34, *p* < 0.05]. Treatment with PGZ led to a significant increase in the expression of pAMPK and pAKT and a decrease in 4E-BP1 compared to the PTZ group. No significant difference was found between groups treated with low and high doses of PGZ.

### 2.5. Pioglitazone Reduced GLUT 1 and GLUT 3 Genes Expression

**Figure 5:**
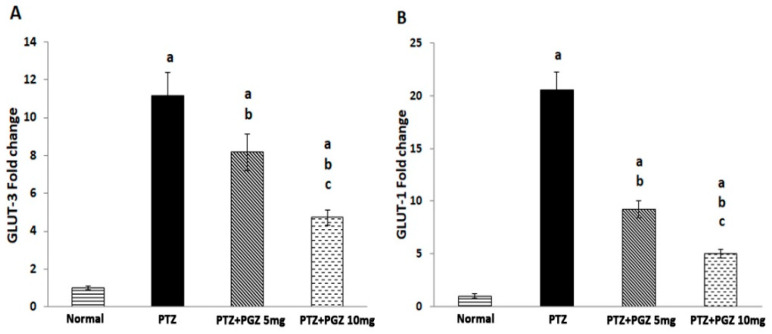
Pioglitazone increased the hippocampal levels of GLUT 1 and GLUT 3 genes in PTZ-kindled mice. (**A**) Mean values of GLUT-3 and (**B**) Mean values of GLUT-1 in hippocampus. Data were expressed as means ± SD. Analysis of quantitative variables was performed using one-way ANOVA followed by Bonferroni’s post-hoc test with *p* < 0.05 considered significant (n = 6). ^a^ significant difference from normal, ^b^ significant difference from PTZ group, ^c^ significant difference from low dose of pioglitazone. GLUT: glucose transporter, PTZ: pentylenetetrazole, PGZ: pioglitazone.

Compared to control animals, GLUT1 expression in the PTZ kindled group increased 20-fold [F (3, 20) = 382.22, *p* < 0.05] and GLUT 3 expression increased 10-fold [F (3, 20) = 1.44, *p* < 0.05]. On the other hand, the two doses of PGZ significantly decreased GLUT-1 and GLUT-3 expressions compared to the non-treated PTZ group (Figure 5).

## 3. Discussion

The current research examined the effect of PGZ, an anti-diabetic drug, in a PTZ-induced seizure model. PTZ was selected to mimic epilepsy as it reliably leads to neuronal excitation and seizure activity. Therefore, it is a successful epileptic agent besides the model being inexpensive and simple to perform [14,15]. Kindling with a sub-convulsive dose of PTZ leads to a chronic model of epilepsy and was used in this study as it is more advantageous over acute models. Spontaneity of seizures and its recurrence are the basic hallmarks of human epilepsy [16]. As such, we employed a model that resembles human epilepsy to search for antiepileptic drugs with novel mechanisms of action [17].

In the current study, histopathological examination of diverse parts of the cortex and hippocampus exhibited an increase in degenerative cells in the PTZ group. Histopathological changes in the brain were found to disrupt neurological functions in epileptic patients [18]. A clinical study that examined brain specimens from patients who underwent surgery to treat intractable epilepsy reported several histopathological changes, mainly hippocampal sclerosis [19].

The hippocampus was selected as an area of interest in evaluating the levels of m-TOR markers, glucose metabolism and NGF. It plays a more essential role in epilepsy than the cortex and amygdala, owing to its adaptability and plasticity [15].

Neurotrophins are believed to have essential roles in the pathophysiology of epilepsy [20]. NGF activates the PI3K/AKT pathway which is important for mTOR activation [21]. Several studies have demonstrated that m-TOR overactivation was associated with epileptogenesis [6,15,22]. In the present research, PTZ kindled mice showed a significant increase in NGF levels compared to controls. This may suggest a possible role of NGF in epileptogenesis. Nevertheless, NGF was also reported to have pro- and/or anti-epileptic effects in several animal models. A study showed a significant decrease in NGF levels in the brain of PTZ-kindled rats [23]. The discrepancies between that study and the present one might be due to the fact that the seizure models are not the same in terms of animals used and different PTZ administration protocols. In the present study PTZ was injected three times a week for five weeks while in the other study PTZ was injected daily for 14 days [23]. Another study demonstrated that blocking the biological activity of NGF retarded seizure progress and inhibited mossy fiber sprouting. In addition, it was found that intravenous administration of NGF accelerated the progression of kindling epileptogenesis and increased mossy fiber sprouting [9].

AMPK, in physiological states, is responsible for Tuberous Complex 2 (TSC2) activation that negatively regulates m-TOR activation, while p-AKT, which lies upstream of m-TOR [24,25], inhibits the TSC2 complex resulting in mTOR activation [26]. In addition, AMPK phosphorylates AKT at its negative regulatory sites [27]. M-TOR regulates protein synthesis through phosphorylation of eukaryotic initiation factor 4E-binding protein (4E-BP1) and 4E-BP1 is a readout of m-TOR [27,28,29]. In the present study, the PTZ group showed m-TOR activation which was evidenced by a decrease in the level of p-AMPK and a rise in 4E-BP1. This was similar to a result from a previous study that demonstrated m-TOR activation in the hippocampus of a PTZ-induced status epilepticus (SE) rat model [15]. The m-TOR pathway contains many feedback loops. The m-TORC1-AKT feedback loop was shown in multiple tissues [30]. An upregulation of the mTORC1/S6K cascade restrains IRS-1 function involving PI3K/AKT activation [30]. This may explain the unpredicted decrease in pAKT levels in the PTZ group.

Seizure induced by PTZ is known to increase cerebral energy demand [31]. Previous studies showed upregulation of both GLUT-1 and GLUT-3 transporter expressions in the brain of PTZ-kindled animals [31,32]. This is in alignment with the current study results.

AMP-activated protein kinase (AMPK) is considered a sensor of changes in cell energy requirements and is stimulated by low energy status resulting from a high ADP/ATP ratio [33]. In the present study, there was an upregulation of both GLUT 1 and GLUT 3 expressions. Therefore, it was predicted that ATP levels increased, which negatively regulated AMPK, and this may explain the decrease in the level of AMPK.

The neuroprotective effects of PPAR-agonists have been demonstrated in various models of central nervous system diseases, including Parkinson’s disease, cerebral ischemia, and Alzheimer’s disease [34,35]. In the present study, it was observed that PGZ diminished the convulsive behavior exhibited by mice in comparison to PTZ-kindled control group. These findings were in agreement with a previous study [13]. There were reductions in degenerated cells in groups receiving PGZ. This finding implies that PGZ exhibited an anti-apoptotic effect in the hippocampi of mice. The current results were in agreement with previous studies that detected a similar effect of PGZ on a kinase-induced, as well as a febrile seizure, model [11,36]. In animal models of tuberous sclerosis complex, mTOR inhibitors were shown to have antiepileptogenic activity. Preliminary clinical studies on patients affected by TSC also showed that mTOR inhibitors reduced seizure activity and were capable of exerting disease modifying effects. Furthermore, studies on genetic and acquired epilepsy models have revealed the antiepileptogenic potential of mTOR inhibitors [4,37,38]. In the current study, PGZ exerted a significant decrease in 4EBP-1, and NGF, and an increase in pAMPK which implies that PGZ could potentially act as an mTOR inhibitor.

M-TOR inhibitors may have antiepileptogenic activity and encourage the autophagic clearance of abnormally folded proteins, but may also interfere with the normal mechanisms of neuroprotection, synaptic plasticity and regeneration. Therefore, identification of key regulators upstream or downstream of the m-TOR pathway may provide more selective therapeutic targets.

The effect of PGZ on glucose transporters in an epilepsy model have not been investigated before. Since glucose metabolism affects AMPK, an upstream mTOR regulator, the present study addressed the effect of PGZ on glucose transporter expression. It has previously been reported that PGZ deceased cerebral glucose utilization in an Alzheimer model in contrast to its stimulatory effect in non-cerebral tissue [39]. These results were similar to the current study where PGZ significantly decreased the GLUT-1 and GLUT-3 transporters compared to the PTZ group. This explains the high level of AMPK in treated groups and hence mTOR downregulation.

In summary, the results of our study point to pioglitazone as a promising candidate in treating epilepsy through antiepileptogenic mechanisms affecting the mTOR pathway. In a previous study, pre-treatment with PGZ decreased neuronal loss that accompanies status epilepticus (SE). Moreover, the results showed that PGZ caused m-TOR downregulation [15]. Collectively, these results indicate that a PPAR- agonist could help in alleviating SE through m-TOR inhibition.

## 4. Materials and Methods

### 4.1. Animals

Swiss male albino mice, weighing 20–25 g, were utilized in the present study. They were obtained from the Serum and Vaccine authority (Cairo, Egypt). Mice housing were in stainless steel cages with dimensions; 50 cm (L) × 30 cm (W) × 30 cm (H) under a normal light/dark cycle at temperature around 25 ± 1 °C and 55–65% relative humidity with free access to water and food. The number of animals were 6 per cage. All animal measures and the experimental protocols were accepted by the research and ethics committee at Faculty of Pharmacy, Suez Canal University (approval no. 201711MA₂) and followed the guidelines of the Canadian Council on Animal Care (CCAC).

### 4.2. Chemicals and Drugs

Pentylenetetrazole (PTZ) was obtained from Sigma Aldrich (St. Louis, MO, USA). For PTZ dissolution, 0.9% sterile saline is used. Pioglitazone (PGZ) was generously provided by Medical Union Pharmaceuticals (Abou Sultan, Ismailia, Egypt) and was dissolved in 0.5% carboxy methyl cellulose (CMC).

### 4.3. Induction of Epilepsy in Mice

Animals were injected with PTZ at a sub-convulsive dose of 35 mg/kg intraperitoneally (i.p.) three times a week for five weeks. The model was considered successful depending on the behavioral performance. Kindling was considered achieved after 14 ± 1 injections or attaining seizure score 4.5 or 5 on three occasions [40].

### 4.4. Experimental Design

Swiss albino mice were randomly distributed into four experimental groups (Six mice per group): Group 1; Control received sterile saline (0.9% NaCl) + (0.5% CMC), Group 2; PTZ control group: received PTZ (35 mg/Kg, i.p.) three times a week for five weeks, Group (3–4): mice received a dose of either 5 or 10 mg/Kg (p.o.) of PGZ dissolved in 0.5% CMC respectively 30 min before each PTZ injection [41]. The selection of PGZ doses was based on a previous study by Hussein et al. [36].

### 4.5. Assessment of Seizure Activity in PTZ-Kindled Mice

The convulsive behavior was observed for half an hour for each mouse after PTZ injection. The analysis of behavioral test was performed by a blinded investigator with no knowledge of the experimental groups. Seizure score was ranked in accordance to Fischer and Kittner scoring system as following: 0—no convulsive activity; 0.5—head nodding; 1—ear and facial spasms; 2—myoclonic jerks; 2.5—frequent clonic forelimb convulsions; 3—severe forelimb clonuses, full rearing; 3.5—rearing and falling with severe forelimb clonus; 4—generalized clonic convulsions with rearing, jumping; 4.5—generalized clonic convulsions with loss of righting reflex; 5—generalized clonic tonic convulsions and status epilepticus. At the end of the study, the mean of seizure scores reported over fifteen injections of PTZ in each group was calculated and compared [42].

### 4.6. Tissue Preparation

At the end of the experiment, mice were sacrificed by cervical dislocation under deep anesthesia using ketamine and xylazine at a dose of (87.5 mg/kg and 12.5 mg/kg i.p.) respectively. Then, the skull was opened through a midline incision on the skin and dorsal neck to uncover the brain. The brain was removed from the cavity and cortical and hippocampal tissues from each group were separated and instantly placed in 10% neutral buffered formalin for fixation. The fixed tissues were subjected to histopathological procedures for staining. The left hippocampus was stored at −80 °C to be used for analysis of biochemical parameters.

### 4.7. Histopathological Examination

For routine histopathological procedures, hippocampal and cortical specimens were dehydrated in an ascending series of ethanol, cleared in xylol and subjected to three changes of paraffin wax. The tissue was sectioned at 6 µm thick sections using a microtome. Staining of the microscopic slides was performed using a hematoxylin and eosin (H&E) stain [43]. The cortical and hippocampal tissue from each section was examined under a light microscope (Olympus Dp25) connected to a digital camera (Olympus Dp25, Tokyo, Japan) used for imaging. Degenerative cells with dark stained pyknotic nuclei and acidophilic cytoplasm were counted from six fields of cortex and hippocampus and averaged for each group. Examination of sections was performed by a blinded investigator with no knowledge of the other data in the experimental groups.

### 4.8. ELISA Analysis

Frozen hippocampus was washed in ice-cold sodium phosphate buffer (PBS). After the brain is blotted, the hippocampus was homogenized ten times in an ice-cold mixture of PBS (NaH_2_PO_4_ and NaHPO_4_, pH 7.4). Afterwards, it was centrifuged at 3000 rpm for 15 min.

NGF levels were assessed using a commercial Enzyme-Linked Immunosorbent Assay (ELISA) kit specific for mice with catalog no.: CSB-E04684m. Absorbances were measured by microplate reader set at 450 nm. NGF levels were expressed as picograms per gram of tissue.

### 4.9. Evaluation of GLUT-1 and GLUT-3 Genes Expression in Hippocampus by Real-Time PCR

Quantitative real-time PCR was used to determine the gene expression of glucose transporters (GLUT-1) and (GLUT-3) in the hippocampal tissue. Total RNA was extracted from hippocampus by SV total RNA isolation system (Promega, Madison, WI, USA) in accordance to the manufacturer’s directions. Nanodrop NA-1000 UV/vis spectrophotometer (Thermo Fisher Scientific Inc. Wilmington, DE, USA) was used to estimate the purity of the concentration of the extracted RNA. Then, the extracted RNA was stored at −80 °C. Results of PCR was gathered using GoTaq^®^ 1-Step RT-qPCR System (Promega, Madison, WI, USA) and the Step One Plus™ Real-Time PCR thermal cycling instrument (Applied Biosystems, Waltham, MA, USA) and using β-actin for data normalization. The thermal cycling consisted of reverse transcription for 15 min at 37 °C, followed by 10 min at 95 °C to inactivate reverse transcriptase, then 40 PCR cycles of the following: denaturation at 95 °C for 10 s, annealing for 30 s and final elongation step at 72 °C for 30 s. The primers and the annealing temperatures used are summarized in Table 1. ∆∆ Ct and fold change for each sample were calculated.

### 4.10. Western Blotting Analysis

The hippocampus was homogenized in lysis buffer containing protease and phosphatase inhibitors to preserve integrity of proteins. The lysates were centrifuged at 16,000× *g* for 10 min. The supernatants were collected and stored at −80 °C. The protein concentrations were determined by the Bradford assay [44]. Western blotting was performed as previously described [27]. Briefly, the lysate was mixed with an equal volume of 2× Laemmli sample buffer and then boiled at 95 °C for 5 min and centrifuged at 10,000× *g* for 10 min. The resulting supernatants were subjected to 12% sodium dodecyl sulfate–polyacrylamide gel electrophoresis. Proteins were transferred to PVDF membranes, using a Bio-Rad Trans-Blot Turbo unit (Bio-Rad Laboratories Ltd., Watford, UK).

The membranes were then incubated with rabbit monoclonal anti-Phospho-AMPKα (Thr172) [Cat#ab 50081, cell signaling technology], rabbit monoclonal anti-Phospho-Akt (Ser473) [Cat#ab 4060 cell signaling technology] or rabbit monoclonal anti-4E-BP1 [Cat#ab 9644, cell signaling technology] as primary antibodies (1:1000 dilution in TBS-T with 5% non-fat milk) at 4 °C overnight. Blots were subsequently washed in TBS-T and incubated with the HRP-conjugated secondary antibody (Goat antirabbit IgG- HRP-lmg Goat mab-Novus Biological, 1:5000 dilution). All incubations were done for 1 h at room temperature. The signals were visualized with chemiluminescence (ClarityTM Western ECL substrate-BIO-RAD, USA cat#170-5060) according to the manufacturer’s protocol and the chemiluminescent signals were captured using a CCD camera-based imager (Figures S1–S4).

### 4.11. Statistical Analysis

Data were statistically analyzed using the SPSS program, version 24 (SPSS Inc., Chicago, IL, USA). All data of the present study were expressed as mean ± standard deviation. Analysis of quantitative variables was done using ANOVA preceded by Bonferroni’s post hoc t-test. Probability values of <0.05 was regarded significant.

## 5. Conclusions

Collectively, the present results suggest that PGZ could be a promising therapeutic to treat seizures associated with epilepsy. Understanding the role of neurotrophins, glucose metabolism and the m-TOR pathway, and their correlations with each other and association with epileptogenesis, may lead to the development of antiepileptic drugs that can treat intractable epilepsy resistant to current AEDs.

## Figures and Tables

**Table 1:** Genes, primer sequences and annealing temperatures of the assessed genes.

Gene	Primers	Annealing Temperature
GLUT-1	Forward: CCAGCTGGGAATCGTCGTT	62 °C
	Reverse: CAAGTCTGCATTGCCCATGAT	
GLUT-3	Forward: CTCTTCAGGTCACCCAACTACGT	55 °C
	Reverse: CCGCGTCCTTGAAGATTCC	
β -actin	Forward: ACGGCCAGGTCATCACTATTG	56 °C
	Reverse: CAAGAAGGAAGGCTGGAAAAGA	

## Data Availability

Data is available within the article and Supplementary Materials.

## Supplementary Materials

The following supporting information can be downloaded at: https://www.mdpi.com/article/10.3390/ph15091113/s1, Figure S1. Western blot of beta actin (loading control); Figure S2. Western blot of Akt; Figure S3. Western blot of 4EBP1; Figure S4. Western blot of AMPK.
