# Supplementary material for: Pioglitazone Ameliorates Hippocampal Neurodegeneration, Disturbances in Glucose Metabolism and AKT/mTOR Signaling Pathways in Pentyelenetetrazole-Kindled Mice
Source: Pharmaceuticals (Basel). 2022 Sep 6;15(9):1113. doi: 10.3390/ph15091113 (PMC9506442; doi:10.3390/ph15091113)

Pioglitazone ameliorates hippocampal neurodegeneration and disturbances in glucose metabolism and AKT/mTOR signalling pathways in Pentylenetetrazole-Kindled mice

Supplementary materials

1. **Figure S1.** Western blot of beta actin (loading control)
2. **Figure S2.** Western blot of Akt
3. **Figure S3.** Western blot of 4EBP1
4. **Figure S4.** Western blot of AMPK

Figure S1. Western blot of beta actin (loading control)

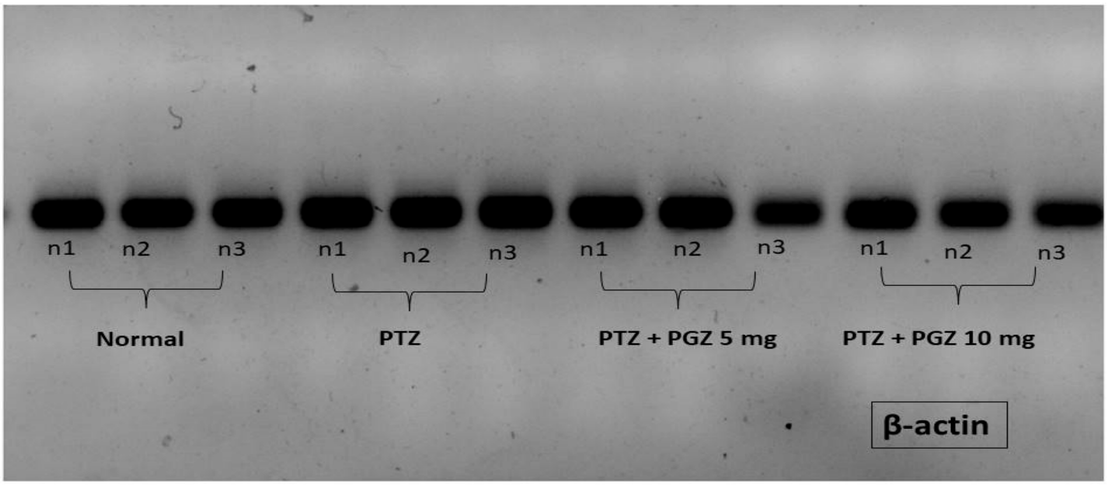

Figure S2. Western blot of Akt

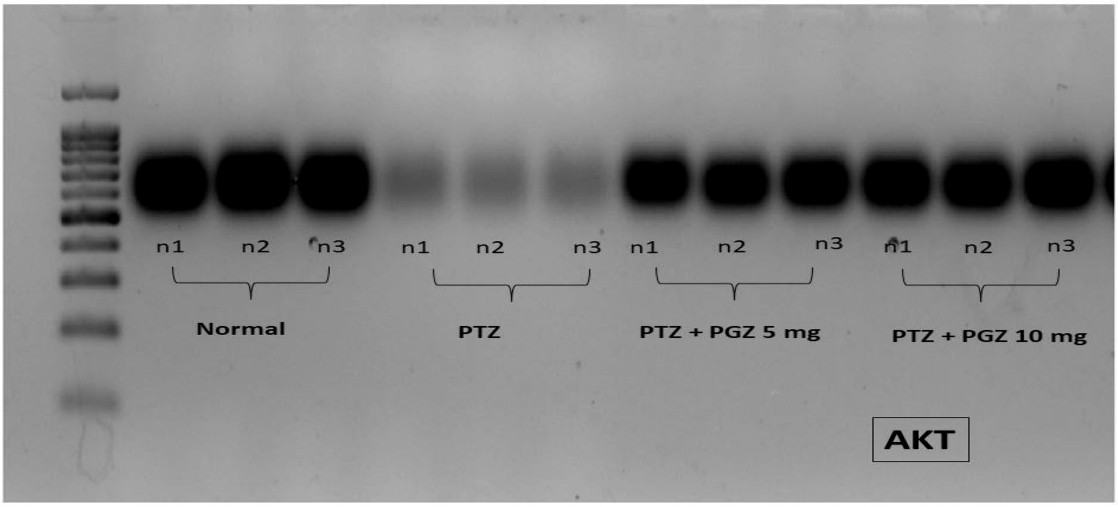

Figure S3. Western blot of 4EBP1

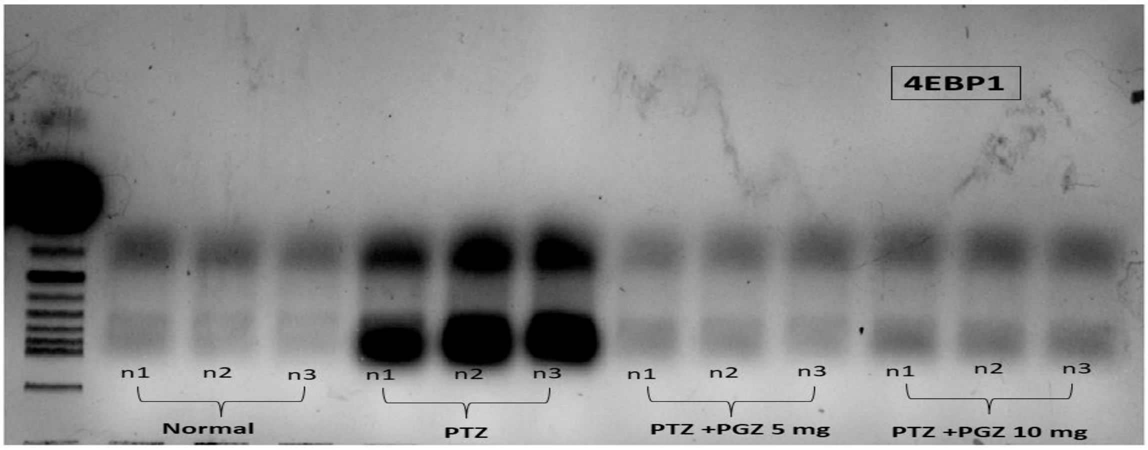

Figure S4. Western blot of AMPK

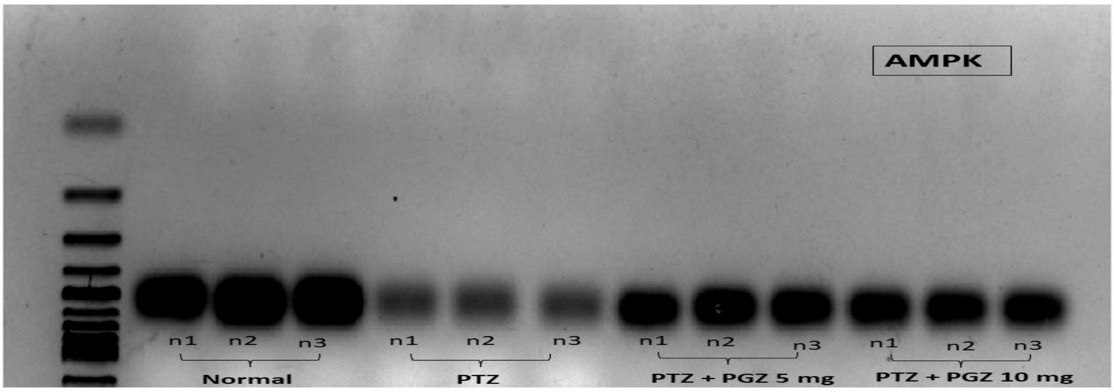

Supplement: Supplementary file 1 [file pharmaceuticals-15-01113-s001.zip › pharmaceuticals-1854941-supplementary.pdf]
